# Supplementary material for: Benefit-cost analysis of raccoon rabies control in Ontario, Canada
Source: PLoS Negl Trop Dis. 2025 Nov 26;19(11):e0013696. doi: 10.1371/journal.pntd.0013696 (PMC12704859; doi:10.1371/journal.pntd.0013696)
Supplement: Attachment — Submitted filename: Shwiff response to reviewers PLoS NTD 2025.docx [file pntd.0013696.s003.docx]

**Author Response:**

Thank you for your careful review of our paper. I have gone through and made the necessary changes and responded to each comment and suggestion individually in blue. The changes in the text are also in blue font for easy identification. These changes have improved our paper, and I appreciate all your feedback. Thank you.

Methods:

-Are the objectives of the study clearly articulated with a clear testable hypothesis stated?

-Is the study design appropriate to address the stated objectives?

-Is the population clearly described and appropriate for the hypothesis being tested?

-Is the sample size sufficient to ensure adequate power to address the hypothesis being tested?

-Were correct statistical analysis used to support conclusions?

-Are there concerns about ethical or regulatory requirements being met?

Reviewer #1: Yes indeed. Objective of the study was well articulated. The study design is also appropriate with sufficient sample size. the issue of ethical clearance was addressed and samples were analysed with proper statically identified software.

Reviewer #2: see general comments

Reviewer #3: The objective of the study is generally clearly articulated and the study design is appropriate.

The end of the final sentence of the intro , "and similar intervention in other areas of North America" is unclear and I think can be deleted. Corrected in text.

In the intro, lines 135 to 145 on p6 may be better suited for the methods or results section. Corrected in text.

More details are needed about the analysis, in particular the perspective and how discounting was implemented. It seems that discounting may not have been used despite the long time horizon of the study - if that's the case, a justification should be provided. Text was added to address this.

I would suggest putting all of the model parameters and assumptions in a table with reference for easy review. We described the model parameters more clearly in text so we did not add an additional figure.

The equations as currently written don't seem correct. In particular, "i" isn't defined, and as currently written, it seems to imply that costs are being summed across scenarios (alpha). Corrected in text.

Some of the next in the methods could be moved to the discussion of limitations (e.g., lines 218-222 on page 11). Corrected in text.

The exclusion of health benefits associated with rabies control is not mentioned (and is a departure from usual CBA methods). The authors should explain why this is the case (lack of data? small expected utility values?), as this will also likely underestimate the full economic value. Corrected in text.

Results lines 260-264 on p. 13 should be moved to the methods. Corrected in text.

An editorial suggestion: it may be more intuitive/logical for readers to put the methods for program costs ahead of the benefits. Corrected in text.

Reviewer #4: This paper reports on an economic analysis of raccoon rabies control in Canada. This is a very useful exercise as we are lacking evidence for the profitability of One Health interventions. However, there are a series of edits and questions to this manuscript, that should be addressed:
It is not clear how the Human Population Risk was validated. This is a limitation of the study that should be stated. Corrected in text.
If I did not overlook it, there is no discounting considered in the BCA. As this is a multi-year exercise, The annual benefits and costs should be discounted with an indicated rate and finally net present values of benefits and cost should be related Corrected in text.

**Results**:

-Does the analysis presented match the analysis plan?

-Are the results clearly and completely presented?

-Are the figures (Tables, Images) of sufficient quality for clarity?

Reviewer #1: The results were well presented. Figures and tables were clearly illustrated.

Reviewer #2: see general comments

Reviewer #3: The data in Fig 3 are also contained in the tables, so could consider removing the figure. Corrected in text.

For Tables 1-3, I think the Incidence and Cost columns represent incidence and costs averted with intervention. If so, consider relabelling to make this clear. Text was added to address this.

Reviewer #4: Figure 3: Turn the text of y-axise to read from bottom to top. It is not clear if the intervention-cost depicted in Figure 3 are empirical or simply model based? I looks like the intervention has an immediate effect on human risk which is rare for mass vaccinations. Hence, the authors should state about the empirical validity of their simulations. In wildlife, re-introductions from outside the intervention area are common. Did the authors consider re-introduction in their model or was this not included. This should be stated. Text was added to address this.

**Conclusions**:

-Are the conclusions supported by the data presented?

-Are the limitations of analysis clearly described?

-Do the authors discuss how these data can be helpful to advance our understanding of the topic under study?

-Is public health relevance addressed?

Reviewer #1: Conclusions were well summarized using the data and the discussions of authors were helpful for other authors and public health concerns.

Reviewer #2: see general comments

Reviewer #3: Would be useful to expand the discussion about the costs and benefits associated with raccoon variant rabies elimination. The authors mention that the cost avoidance savings accrue into perpetuity, but are there also ongoing costs associated with maintaining elimination? Text was added to address this.

Although a range of scenarios are presented, can the authors provide some commentary on what scenarios are considered more/or less probable? i.e., are all considered equally probable or is one more probable? We are reluctant to give an opinion regarding the probability of which scenario is more likely because of lack of data.

Reviewer #4: Finally, the authors should refer to comparable studies and new methods, using for example game-theoretical strategy analysis and human capital benefits [1]. Text was added to address this.

1. Bucher, A., Dimov, A., Fink, G., Chitnis, N., Bonfoh, B., Zinsstag, J., Benefit-cost analysis of coordinated strategies for control of rabies in Africa. Nature Communications, 2023. 14: p. 5370.

**Editorial and Data Presentation Modifications?**

Use this section for editorial suggestions as well as relatively minor modifications of existing data that would enhance clarity. If the only modifications needed are minor and/or editorial, you may wish to recommend “Minor Revision” or “Accept”.

Reviewer #1: No comment

Reviewer #2: see general comments

Reviewer #3: The abstract includes information that is not included in the rest of the manuscript (scenario 2) and focus more on summarizing the epidemiological modelling that informs the CBA. The abstract should be revised to focus on the methods used in the current study. Corrected in text.

Reviewer #4: I made suggestions for edits of the tables and figures Corrected in text.

**Summary and General Comments**:

Use this section to provide overall comments, discuss strengths/weaknesses of the study, novelty, significance, general execution and scholarship. You may also include additional comments for the author, including concerns about dual publication, research ethics, or publication ethics. If requesting major revision, please articulate the new experiments that are needed.

Reviewer #1: The cost benefit analysis study topic has significant contribution as more studies and publications have not clearly work on cost benefit analysis of a given subject of study.

Reviewer #2: General comments:

Applying economics to support decision-making in the fight against animal diseases is becoming increasingly important. This applies in particular to the control of wildlife diseases, where the benefits of the measures are often questioned. The paper by Shwiff et al. deals with a benefit-cost analysis to examine the economic benefits of intervention strategies in the frame Canada’s largest raccoon rabies outbreak in history which began in 2015.

To this end they used an epidmiological disease spread model and compared three different intervention strategies with scenario 1 and 2 estimating the spread of the raccoon rabies variant if control measures were continued until 2025 or ended in 2020, respectively, and scenario 3 as a no-intervention strategy. Results from this analysis indicated savings for Ontario ranging from $53 million to $495 million CAD over the study period (2015 to 2025) compared to a no-intervention strategy depending on low, medium, or high estimates of each of the variables.

This is interesting information that is worth publishing, however, one cannot help but get the impression that the paper was written in a rush. The paper sometimes lacks the diligence required for publication, but this can be easily remedied. To be clear, there is no doubt about the methodology and modeling used, it is rather the uninspiring, mathematically undercooled presentation. One would have liked a more interesting and vivid presentation of the manuscript for the reader. A little bit more of information and input would give the reader a better picture. Text was added to address this.

Here some points to consider for improving the manuscript.

• The introduction section is less inspiring and informative. A brief introduction to the topic (the first author herself wrote an nice article on the topic - Shwiff et al., 2018) and mentioning of published literature on rabies specific cost-benefit analyses would be desirable. It would also be good to briefly describe what concrete measures have been taken since 2015 to contain raccoon rabies by referring to published literature instead of focusing extensively on the agent-based model and roles and responsibilities of different players. Text was added to address this.

• Often references are missing and should be provided whenever needed (see lines 111, 113, 178, . Corrected in text.

• Lines 182-189: This part is a little confusing. Can the AT and INVT rates from a area endemic for dogs rabies be compared one-to-one with those in an area endemic for raccoon rabies (see reference 17)? These are two different scenarios. In the former the biting incidence per 100,000 inhabitants and the resulting PEP can be much higher. Two sentences later it is stated that data were sourced on the range of annual case frequency of PEP and AT during raccoon rabies epizootics in the Eastern United States and Eastern Canada as proxies. How does this fit with the information provided above? Corrected in text.

• Lines 217-222: Would this part not better suited for the Discussion section? Corrected in text.

• Line 227: We are now in the year 2025, so “forecasted” sounds a bit strange. The costs for 2023 and 2024 should actually be available and for this year we should know what is to be spent. Would it be possible to provide a annual breakdown of costs? Corrected in text.

• Line 229: What costs are included here, e.g. costs for TVR, ORV (vaccine price, costs for bait distribution, transport, storage…) etc.. Is it direct costs only? Corrected in text.

• Line 250: There is no mention of the total population of Ontario and in particular the population living in the area shown in Figures 1 and 2. The HRP appears to be quite high. From a practical perspective, it is hard to imagine that 75% of the population in Ontario would be at risk in a no-intervention scenario.... Could it be that the modeling assumptions are a bit biased? Could this be discussed under the limitations of the study? Corrected in text.

• Lines 260-264: This belongs to Materials & Methods. Corrected in text.

• Lines 270-273, 275-277, 279-281: For the three tables always the same text wording is used. Corrected in text.

• Discussion: This part needs improvements. The significance, importance and relevance of the results obtained should be objectively discussed against available literature on the topic (Meltzer, 1996; Elser et al., 2016; Bucher et al., 2023). The limitations of the study should more clearly discussed (see above). Text was added to address this.

• Figures: the captions are generally very poor. Corrected in text.

• Figure 1 and 2: These do not appear to be original figures. Please provide the source or describe how they were made. Also, only the southeasternmost part of Ontario is shown in the figures. Since not everyone is familiar with the topography, a map of Ontario with an enlargement of the area under study would be desirable so that the unfamiliar reader can orient himself better. Corrected in text.

• Figure 3: The y-achsis should be adjusted to zero. Corrected in text.

• Tables 1-3: There is redundant information. The figures in the first three columns of each table are already shown graphically in Figure 3 and can be removed. When the number of population saved (column 4) could be included in Figure 3 as well, than all three tables could be combined in one only showing the outcome if different levels of benefit are applied. Corrected in text.

• Table 4 is redundant as it is just a summary of Tables 1-3. Corrected in text.

Reviewer #3: This is a valuable study that assesses the cost-benefit of raccoons rabies control in Ontario, Canada. Quantifying the value of prevention is important for ensuring ongoing support for programs such as this. Strengths of this analysis include the use a previously published epidemiologic model to develop a "no intervention" counterfactual scenario and consideration of a range of outcomes using data from other jurisdictions. Overall, the study would be strengthened by more details about the economic analysis.

Reviewer #4: This paper merits publication if the methodological questions, mainly of the discounting are resolved. Corrected in text.

PLOS authors have the option to publish the peer review history of their article ([what does this mean?](https://gcc02.safelinks.protection.outlook.com/?url=https%3A%2F%2Ftrack.editorialmanager.com%2FCL0%2Fhttps%3A%252F%252Fjournals.plos.org%252Fplosntds%252Fs%252Feditorial-and-peer-review-process%2523loc-peer-review-history%2F1%2F010f019518be2985-ca9afb71-9a4f-49bf-8194-f4cc9aaeda0e-000000%2FsRiYW1Vrij5zJmyiuNVHdH3GITtIgvJ_CG5Yj3UfdS8%3D198&data=05%7C02%7Cstephanie.a.shwiff%40usda.gov%7Cb178a795cfe64a6fb9f308dd737640a9%7Ced5b36e701ee4ebc867ee03cfa0d4697%7C1%7C0%7C638793674522248827%7CUnknown%7CTWFpbGZsb3d8eyJFbXB0eU1hcGkiOnRydWUsIlYiOiIwLjAuMDAwMCIsIlAiOiJXaW4zMiIsIkFOIjoiTWFpbCIsIldUIjoyfQ%3D%3D%7C0%7C%7C%7C&sdata=K0E6bHjY8pgs9l3xWBa3%2BDIsMxQj%2F35NP7zntRWuo6o%3D&reserved=0)). If published, this will include your full peer review and any attached files.

If you choose “no”, your identity will remain anonymous but your review may still be made public.

**Do you want your identity to be public for this peer review?** For information about this choice, including consent withdrawal, please see our [Privacy Policy](https://gcc02.safelinks.protection.outlook.com/?url=https%3A%2F%2Ftrack.editorialmanager.com%2FCL0%2Fhttps%3A%252F%252Fwww.plos.org%252Fprivacy-policy%2F1%2F010f019518be2985-ca9afb71-9a4f-49bf-8194-f4cc9aaeda0e-000000%2Ffg4VF5S6EwAZblAL3_duGFO4gRJN3bn48lqn1odcmeI%3D198&data=05%7C02%7Cstephanie.a.shwiff%40usda.gov%7Cb178a795cfe64a6fb9f308dd737640a9%7Ced5b36e701ee4ebc867ee03cfa0d4697%7C1%7C0%7C638793674522261318%7CUnknown%7CTWFpbGZsb3d8eyJFbXB0eU1hcGkiOnRydWUsIlYiOiIwLjAuMDAwMCIsIlAiOiJXaW4zMiIsIkFOIjoiTWFpbCIsIldUIjoyfQ%3D%3D%7C0%7C%7C%7C&sdata=0r9sZm3G12I7FtDMQ9rNQlJVra5HH7hDp9SWlkUTT8E%3D&reserved=0).

Reviewer #1: No

Reviewer #2: No

Reviewer #3: No

Reviewer #4: No

[NOTE: If reviewer comments were submitted as an attachment file, they will be attached to this email and accessible via the submission site. Please log into your account, locate the manuscript record, and check for the action link "View Attachments". If this link does not appear, there are no attachment files.]

**Figure resubmission:**

While revising your submission, please upload your figure files to the Preflight Analysis and Conversion Engine (PACE) digital diagnostic tool, [https://pacev2.apexcovantage.com/](https://gcc02.safelinks.protection.outlook.com/?url=https%3A%2F%2Fpacev2.apexcovantage.com%2F&data=05%7C02%7Cstephanie.a.shwiff%40usda.gov%7Cb178a795cfe64a6fb9f308dd737640a9%7Ced5b36e701ee4ebc867ee03cfa0d4697%7C1%7C0%7C638793674522273874%7CUnknown%7CTWFpbGZsb3d8eyJFbXB0eU1hcGkiOnRydWUsIlYiOiIwLjAuMDAwMCIsIlAiOiJXaW4zMiIsIkFOIjoiTWFpbCIsIldUIjoyfQ%3D%3D%7C0%7C%7C%7C&sdata=TBI5yUwPTRqP1IZ%2FaEzRHfjqZ%2BpiSLqPcxlTOb6OWHo%3D&reserved=0). PACE helps ensure that figures meet PLOS requirements. To use PACE, you must first register as a user. Registration is free. Then, login and navigate to the UPLOAD tab, where you will find detailed instructions on how to use the tool. If you encounter any issues or have any questions when using PACE, please email PLOS at [figures@plos.org](mailto:figures@plos.org). Please note that Supporting Information files do not need this step. If there are other versions of figure files still present in your submission file inventory at resubmission, please replace them with the PACE-processed versions.

**Reproducibility:**

To enhance the reproducibility of your results, we recommend that authors of applicable studies deposit laboratory protocols in protocols.io, where a protocol can be assigned its own identifier (DOI) such that it can be cited independently in the future. Additionally, PLOS ONE offers an option to publish peer-reviewed clinical study protocols. Read more information on sharing protocols at [https://plos.org/protocols?utm_medium=editorial-email&utm_source=authorletters&utm_campaign=protocols](https://gcc02.safelinks.protection.outlook.com/?url=https%3A%2F%2Fplos.org%2Fprotocols%3Futm_medium%3Deditorial-email%26utm_source%3Dauthorletters%26utm_campaign%3Dprotocols&data=05%7C02%7Cstephanie.a.shwiff%40usda.gov%7Cb178a795cfe64a6fb9f308dd737640a9%7Ced5b36e701ee4ebc867ee03cfa0d4697%7C1%7C0%7C638793674522285875%7CUnknown%7CTWFpbGZsb3d8eyJFbXB0eU1hcGkiOnRydWUsIlYiOiIwLjAuMDAwMCIsIlAiOiJXaW4zMiIsIkFOIjoiTWFpbCIsIldUIjoyfQ%3D%3D%7C0%7C%7C%7C&sdata=oJAfgisI21aBlPrEhxv6goYbnWNYSEF8j5FawSSt%2FT8%3D&reserved=0)
