# Supplementary material for: Benefit-cost analysis of raccoon rabies control in Ontario, Canada
Source: PLoS Negl Trop Dis. 2025 Nov 26;19(11):e0013696. doi: 10.1371/journal.pntd.0013696 (PMC12704859; doi:10.1371/journal.pntd.0013696)
Supplement: Attachment — Submitted filename: Rebuttal - Response to Reviewers 9.2.25.docx [file pntd.0013696.s004.docx]

Stephanie Shwiff
National Wildlife Research Center
4101 Laporte Ave

Fort Collins, CO 80521
stephanie.a.shwiff@usda.gov

September 4, 2025

Dear Reviewers,

I am pleased to re-submit our manuscript titled "Benefit-Cost Analysis of Raccoon Rabies Control in Ontario, Canada" for consideration for publication in PLoS Neglected Tropical Diseases. Thank you for a second round of review and comments of our paper. I apologize that some previous specific comments and changes were lost in the major re-writes that the paper experienced in the first round of editing. I have gone through and made the necessary changes and responded to each comment and suggestion individually in blue (within this document, not the manuscript). These changes continue to improve our paper, and I appreciate all your feedback. Below is the line by line response to each reviewer comment:

**Methods**

Reviewer #2: Yes, all of the reviewers' comments were addressed appropriately.

Reviewer #3: (No Response)

Reviewer #4: In there letter of revision the authors claim that the changes in the revised manuscript are in blue. But I don’t see any such changes. I am afraid that they uploaded the wrong manuscript. I can not judge the reivsions if I don’t see the revised manuscript.
I would also appreciate if the authors respond to the query in the letter of revision and not only mention that a change is made in the text.

We have updated responses for Reviewer 4 and have also updated the 5/2 comments and updated revision responses, referring to specific line changes. Response to reviewers and revised manuscript with track changes were uploaded to the portal on 5/1 and 5/2 – however they for some reason are not checked under “display on TF” so perhaps the reviewer was unable to view them – apologies! Hopefully this time around you will be able to access the following submitted files:

**Rebuttal Letter - Response to Reviewers 9.2.25 PNTD-D-24-01269R1.docx**

**Revised Manuscript with Track Changes 9.2.25 PNTD-D-24-01269R1.docx**

**BCA of racoon rabies control in Ontario, Canada Shwiff 9.2.25 PNTD-D-24-01269R1.docx**

**Response to Reviewers 5.2.25 PNTD-D-24-01269R1.docx**

If I did not overlook it, there is no discounting considered in the BCA. As this is a multi-year exercise, The annual benefits and costs should be discounted with an indicated rate and finally net present values of benefits and cost should be related

Corrected in text. Line 196-199. Supplementary materials added for reviewers as well (excel spreadsheet). Now reads:

Based on publicly available data from the Bank of Canada, an average rate of inflation for the study period of 3% was utilized [31]. For this analysis we incorporated a 3% real discount rate into the estimation of benefits and costs accrued over the period to compare benefits and costs over the lifetime of the project.

**Results**

Reviewer #2: Yes, all of the reviewers' comments were addressed appropriately.

Reviewer #3: I may have missed it, but I don't see the program costs presented in the results, just the savings.

Since this is a modeling study the costs are a component of the methods, used to calculate the savings, which are the primary result.

Line 268 - Ontario PEP costs used for this analysis were $2,793 CAD including indirect costs ($2,100 direct + $693 indirect) (H. McClinchy, personal communication, December 1, 2023). Cost for AT was estimated at $300 CAD and INVT was $200 CAD for the analysis [3]. The number of individuals protected or ‘saved’ by the program was then combined with the rates of incidence of PEP, AT and INVT per 100,000 people to monetize the benefits of the program.

Reviewer #4: (No Response)

**Conclusions**

Reviewer #2: Yes, all of the reviewers' comments were addressed appropriately.

Reviewer #3: (No Response)

Reviewer #4: (No Response)

**Editorial and Data Presentation Modifications**

Reviewer #2: Accept

Reviewer #3: For the intro, I found the paragraph on p. 6 (lines 122 to 133) hard to follow in terms of the logic. The emphasis on gaps in high-risk, low-income settings seems less directly relevant to this study on raccoon rabies in a high-income context. I also wasn't sure how the mention of game theoretic methods fits in.

While not directly relevant, this is important information to note in the introduction to give readers context on the current availability of rabies economic research. The mention of game theory is a forward-looking suggestion for future research, providing an example of a more advanced analytical method – added per Reviewer #4’s recommendation.

Reviewer #4: (No Response)

**Summary and General Comments**

Reviewer #2: The time the authors have spent responding to the reviewers' comments has definitely paid off. The quality of the manuscript has improved significantly; the work is now worthy of publication.

Reviewer #3: It is difficult to assess whether the specific reviewer comments were addressed, as the authors did not provide a response document mapping their revisions to the reviewer feedback. Additionally, the tracked changes version is not helpful for this purpose, since it shows the entire text as replaced, making it unclear where specific changes were made. I also note that the query regarding the use of discounting does not appear to have been addressed.

I apologize for the inconvenience caused by the large-scale rewrites, which I understand made it difficult to pinpoint specific changes based on the feedback. I hope efforts to address your feedback are more transparent in this round of revisions with the submitted files detailed above. The discount rate has now been explicitly addressed. I have updated the text on Line 196-199 to clarify that a discount rate was included in the analysis (not clearly stated in the previous version of the methods section). Thank you for pointing out this omission.

Reviewer #4: (No Response)

We look forward to your feedback and hope that our manuscript will contribute to the ongoing dialogue and research efforts within the NTD community. Thank you for your continued efforts our polishing our submission.

Sincerely,

Stephanie Shwiff
